# Impacts of Plant-Based Foods in Ancestral Hominin Diets on the Metabolism and Function of Gut Microbiota *In Vitro*

**DOI:** 10.1128/mBio.00853-14

**Published:** 2014-05-20

**Authors:** Gary S. Frost, Gemma E. Walton, Jonathan R. Swann, Arianna Psichas, Adele Costabile, Laura P. Johnson, Matt Sponheimer, Glenn R. Gibson, Timothy G. Barraclough

**Affiliations:** ^a^ Nutrition and Dietetic Research Group, Section of Investigative Medicine, Division of Diabetes, Endocrinology and Metabolism, Imperial College London, Hammersmith Hospital, London, United Kingdom; ^b^ Food Microbial Sciences Unit, School of Chemistry, Food and Pharmacy, University of Reading, Reading, United Kingdom; ^c^ Department of Life Sciences, Imperial College London, Ascot, Berkshire, United Kingdom; ^d^ Department of Anthropology, University of Colorado at Boulder, Boulder, Colorado, USA

## Abstract

Ancestral human populations had diets containing more indigestible plant material than present-day diets in industrialized countries. One hypothesis for the rise in prevalence of obesity is that physiological mechanisms for controlling appetite evolved to match a diet with plant fiber content higher than that of present-day diets. We investigated how diet affects gut microbiota and colon cells by comparing human microbial communities with those from a primate that has an extreme plant-based diet, namely, the gelada baboon, which is a grazer. The effects of potato (high starch) versus grass (high lignin and cellulose) diets on human-derived versus gelada-derived fecal communities were compared *in vitro*. We especially focused on the production of short-chain fatty acids, which are hypothesized to be key metabolites influencing appetite regulation pathways. The results confirmed that diet has a major effect on bacterial numbers, short-chain fatty acid production, and the release of hormones involved in appetite suppression. The potato diet yielded greater production of short-chain fatty acids and hormone release than the grass diet, even in the gelada cultures, which we had expected should be better adapted to the grass diet. The strong effects of diet on hormone release could not be explained, however, solely by short-chain fatty acid concentrations. Nuclear magnetic resonance spectroscopy found changes in additional metabolites, including betaine and isoleucine, that might play key roles in inhibiting and stimulating appetite suppression pathways. Our study results indicate that a broader array of metabolites might be involved in triggering gut hormone release in humans than previously thought.

## IMPORTANCE

One theory for rising levels of obesity in western populations is that the body’s mechanisms for controlling appetite evolved to match ancestral diets with more low-energy plant foods. We investigated this idea by comparing the effects of diet on appetite suppression pathways via the use of gut bacterial communities from humans and gelada baboons, which are modern-day primates with an extreme diet of low-energy plant food, namely, grass. We found that diet does play a major role in affecting gut bacteria and the production of a hormone that suppresses appetite but not in the direction predicted by the ancestral diet hypothesis. Also, bacterial products were correlated with hormone release that were different from those normally thought to play this role. By comparing microbiota and diets outside the natural range for modern humans, we found a relationship between diet and appetite pathways that was more complex than previously hypothesized on the basis of more-controlled studies of the effects of single compounds.

## INTRODUCTION

Obesity, the health consequences of obesity, and the associated morbidity are major public health issues for western populations (1). The increased prevalence of obesity has been driven by changes in diet and energy expenditure (2). Energy expenditure has reduced across Europe due to a number of factors, including the reduction in manual labor and the mechanization of transport (3). Evidence suggests that energy intake (composed of a high percentage of highly palatable and high-energy dense foods) has not fallen by the same magnitude, leading to a mismatch between energy expenditure and diet that drives the current obesity epidemic (4).

Diet has changed rapidly across the western world in the last 100 years. “Fast food,” which packs palatable food into convenient servings that can be prepared and eaten quickly, is popular across Europe and North America and has an increasing market size. Such foods have high energy, low fiber, and high fat content (5). There is evidence that energy-dense foods produce lower satiety and satiation signals than equivalent low-energy foods (6). This diet is markedly different from the historical low-energy-density, nutrient-poor diet that the human gut was adapted to over several millennia (7). Insight into how appetite responds to modern diets may be gained by studying the effect of ancestral-type, nutrient-poor diets.

Paleontological evidence supports the idea that some early hominins consumed much more plant material than present-day humans. For example, in *Paranthropus boisei* (*P. boisei*), an early hominin, results of stable-isotope and microwear analyses are consistent with a diet dominated by C_4_ plants (mostly tropical grasses and sedges [8]). However, morphological traits of *P. boisei*, such as robust mandibles, large cheek teeth, and thick dental enamel, suggest that their diet also contained hard foods such as seeds or roots and tubers. It is likely that such diets generated large amounts of materials, such as lignin and cellulose, that escape digestion in the upper gut. Much of this material can be fermented by the colonic microbiota to a number of products, including short-chain fatty acids (SCFA). This process serves as an energy harvest system for undigested material, rescuing energy that cannot be absorbed in the small bowel (9). For example, lowland gorillas derive around 57% of their metabolizable energy from SCFA, compared to 1.2% in the current western human diet (10). A shift occurred in the genus *Homo* toward more varied diets, incorporating a mixture of hard and soft plant foods and animal tissues (11). Yet it remains certain that for most of history, the human lineage consumed more indigestible plant material than the present western-style diet.

One proposed explanation for the rise in obesity, therefore, is that there is a mismatch between the composition of recent European and North American diets and physiological mechanisms for suppressing appetite (which evolved in response to ancestral diets). Of particular interest is the recent description of a large number of nutrient-sensing G protein-coupled receptors on the apical surface of the neuroendocrine L cell (12). This cell secretes anorectic gut hormones such as peptide YY (PYY) and glucagon-like-peptide-1 (GLP-1) (13). Such hormones also have major effects on gastric motility, with increasing concentrations causing a decrease in gastric emptying and slowing small-intestinal transit (14), therefore increasing the efficiency of absorption. The best-characterized ligands for the G protein receptors free fatty acid receptor 2 (FFAR2) and FFAR3 (FFAR2/3) are SCFA produced by the fermentation of fiber (15), and there is now clear evidence that suggests that increased SCFA production in the colon increases the release of PYY and GLP-1 (16, 17). The L cell also expresses receptors for bile acids, medium- and long-chain fatty acids, and amino acids, which all stimulate PYY and GLP-1 (18).

One species of modern primate that feeds on particularly nutrient-poor plant material is the gelada baboon (*Theropithecus gelada*). Native to the highlands of Ethiopia, these animals subsist largely as grazers, the only modern primate species to do so. The remains of their fossilized ancestors displayed isotope ratios indicative of C_4_ plant dietary components equivalent to those seen with some lineages of early hominins (8). If gut microbiota are adapted to the diet of their host, we would expect the microbiota of gelada baboons to be efficient at extracting energy from their grass-based diet (19). Comparing the capacities of gelada microbiota and human microbiota to extract energy from energy-poor diets and to generate metabolites that feed into gut hormone pathways might provide insights into the potential consequence of energy-poor diets in ancestral hominins for gut signaling pathways.

To determine the role of the gut microbiome in host appetite responses to dietary components and establish if such responses are optimized to the typical diet of their host, we compared potato and grass diets fermented by human and gelada microbiota *in vitro*. Using a fluorescence in-situ hybridization (FISH) approach, we characterized diet-induced shifts in the populations of bacterial groups whereas the functionality of the microbiota was assessed using a combination of targeted and untargeted metabolic profiling techniques. Specifically, SCFA concentrations were measured by gas chromatography (GC) and the global metabolite signatures were obtained using a ^1^H nuclear magnetic resonance (NMR)-based metabonomic approach. The impact of these microbial products on host appetite responses was determined by assaying PYY production in an established mouse colonic cell model. By comparing microbiota and diets outside the natural range for modern humans, our results point to a more complex relationship between diet and appetite suppression pathways than previously hypothesized on the basis of more-controlled studies of single metabolites.

## RESULTS

Anaerobic batch culture fermenters were inoculated with fecal samples from three vegetarian human volunteers and three gelada baboon individuals. For each volunteer, 3 cultures were set up: one on a control diet containing low concentrations of indigestible carbohydrates; one on a high-starch diet consisting of potato predigested to mimic the action of digestion in the stomach and small intestine; and one consisting of grass predigested to mimic the action of the stomach and small intestine. Raw potato contains ~60% starch and 9% dietary fiber (dry weight; USDA nutritional database), whereas *Festuca* leaves contain negligible starch and around 60% nonstarch fiber (lignin, cellulose, and hemicellulose) (20). The mass of predigested material was adjusted so that the total amount of indigestible fiber was consistent for both dietary treatments, with only the fiber types differing.

### Differences between human and gelada gut communities and the effects of diet on these communities.

**FIG 1:**
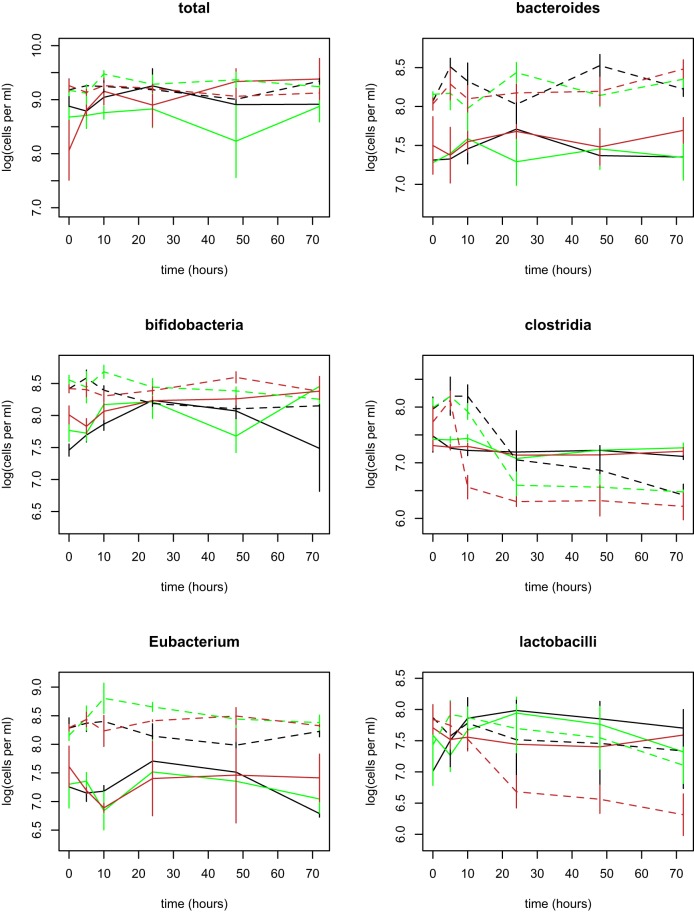
The mean densities of total bacteria and each of the five focal taxa during the experiment. Colors indicate diet: black = control; green = grass; brown = potato. Solid lines = gelada cultures. Dashed lines = human cultures. Standard errors are shown. All taxa were significantly more abundant in human cultures (all *t* > 3, all *P* < 0.005) except lactobacilli (*t* = 1.0, *P* = 0.32).

We estimated the densities of total bacteria and 5 functionally important taxa using FISH (Materials and Methods; see Table S1 in the supplemental material for a description of the taxonomic range covered by each primer set). We chose FISH to focus on key taxa of interest and to obtain numerical counts. We also note that, in the case of bifidobacteria, an important taxon degrading plant starch and other fibers, it was recently reported that standard primers used in 16S sequencing led to underrepresentation of this genus (21). This was another reason for choosing FISH focused on taxa we thought *a priori* might be important for shaping responses. Human-derived cultures contained on average five times more bacterial cells than the gelada-derived cultures at time 0 (linear model, *t* = 2.9, *P* = 0.01; Fig. 1; see also Fig. S1 in the supplemental material). This included six times as many *Bacteroides* bacteria and relatives (*t* = 6.1, *P* < 0.0001), 10 times as many *Eubacterium rectale* bacteria and relatives (*Clostridium* group XIVa; *t* = 4.5, *P* = 0.0003), five times as many bifidobacteria (*t* = 6.4, *P* < 0.0001), three times as many bacteria of clostridium groups I and II (*t* = 3.3, *P* = 0.005), and twice as many lactobacilli (the results for the latter did not reach statistical significance; *t* = 1.0, *P* = 0.32; Fig. 1).

The total density of bacteria in the gelada-derived cultures increased over time such that from 24 h on these cultures no longer contained fewer total bacteria than the human-derived cultures (*t* < 1.1, *P* > 0.2 for each time point; Fig. 1). Diet had no effect on the total density of bacteria in either source community (*F*_2,103_ = 0.62, *P* > 0.5). Densities of members of the *Bacteroides* and those of the *E. rectale* taxa both remained higher in human cultures than in gelada cultures and showed no significant trends with time or diet (Fig. 1). Numbers of clostridium groups I and II, bifidobacteria, and lactobacilli all declined in human cultures over time (interaction between time and community, *t* = −6.9, *P* < 0.0001 in clostridia; *t* = −2.8, *P* = 0.006 in bifidobacteria; *t* = −3.2, *P* = 0.002 in lactobacilli), especially on the potato diet for clostridia and lactobacilli (Fig. 1). None of the taxa declined in numbers in the gelada cultures. From 24 h on, the numbers of bifidobacteria did not differ significantly between human and gelada cultures, whereas the numbers of clostridia were now lower in human cultures than in gelada cultures, with similar results for lactobacilli on the potato diet.

**FIG 2:**
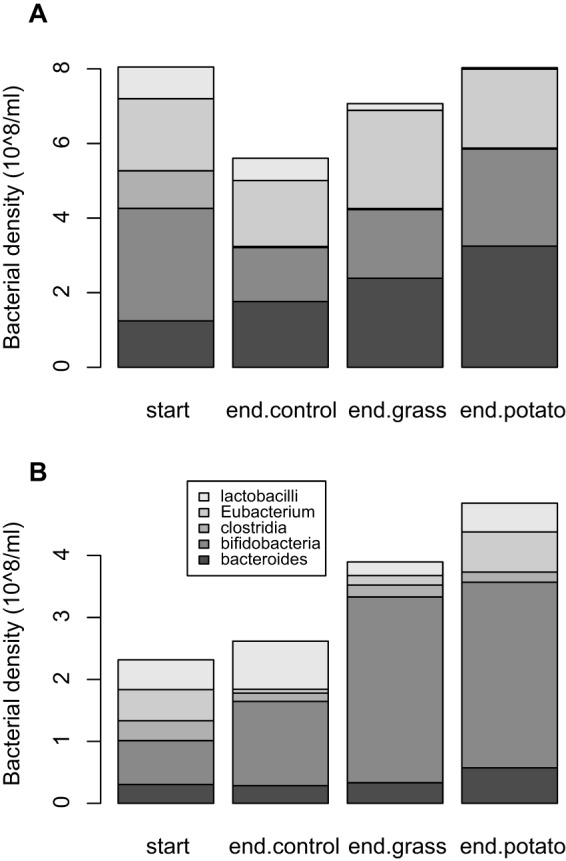
Bacterial density (cells/ml) of five focal taxa enumerated using FISH. Compositions are shown for the initial community and for each dietary control at the end of the experiment (72 h) for human-derived cultures (A) and gelada-derived cultures (B) in turn.

Principal component analysis confirmed that the main sources of variation in taxonomic composition were (i) higher densities of *Bacteroides*, *E. rectale*, and (initially) bifidobacteria in human versus gelada communities and (ii) decreasing densities of clostridium groups I and II and lactobacilli in human cultures over time, especially on potato (see Fig. S2 in the supplemental material). The net effect was a higher relative abundance of members of the *Bacteroides* and *E. rectale* taxa and lower abundance of clostridium groups I and II, bifidobacteria, and lactobacilli in human cultures than in gelada cultures by the end of the experiment, especially on the potato diet (Fig. 2).

### Effects of diet, source community, and bacterial taxa on SCFA production.

**FIG 3:**
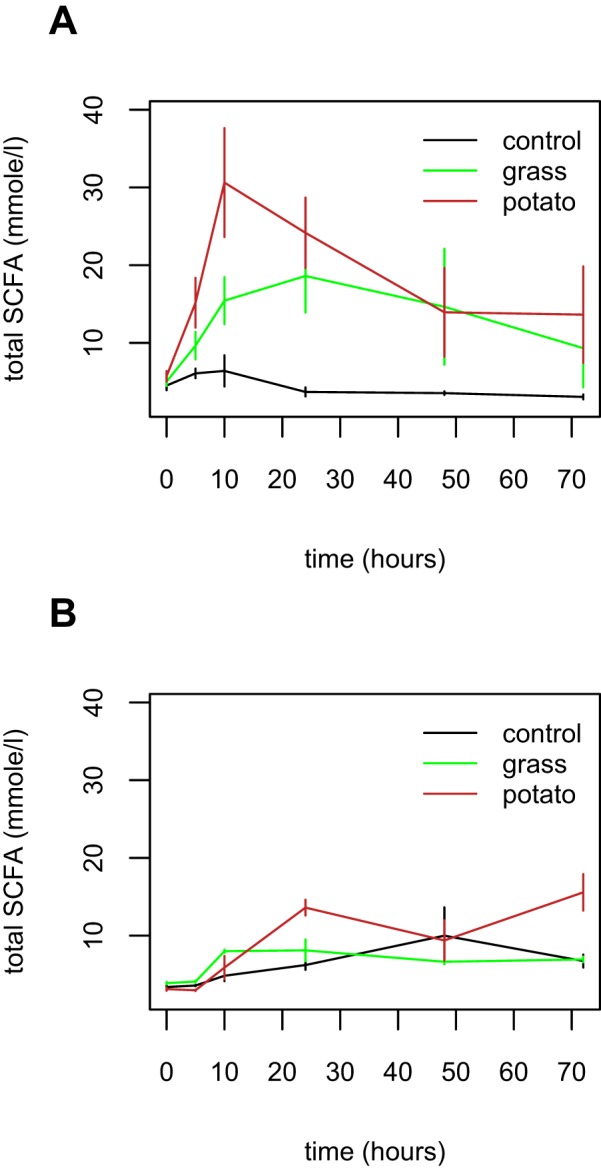
Total short-chain fatty acid production over time in human (A) and gelada (B) cultures. Black = control diet; brown = potato-supplemented diet; green = grass-supplemented diet.

Acetate dominated the SCFA profiles (between 61% and 100%; mean across samples, 93%). To investigate the total production of major unbranched SCFA, the concentrations of acetate, butyrate, and propionate in the cultures were summed. Human-derived cultures had higher levels of total SCFA (and all three main SCFA) than gelada-derived cultures at time 0 (all *t* > 2.55, *P* < 0.05). The total SCFA concentration increased and then decreased over time (time^2^, *F*_1,99_ = 11.7, *P* = 0.0009; Fig. 3). The greatest production of SCFA was observed on the potato diet followed by the grass diet and then the control diet (*F*_2,99_ = 15.9, *P* < 0.0001). The effects of diet and time were greater in the human cultures than in the gelada cultures (*F*_2,99_ = 7.2 and *P* = 0.001 and *F*_1,99_ = 6.8 and *P* = 0.011, respectively): human cultures produced higher peak SCFA concentrations on both grass and potato diets. However, by 72 h, only diet had a significant effect (*F*_2,15_ = 4.8, *P* = 0.024).

The highest concentrations of SCFA were observed in human cultures on the potato diet. There was no evidence for higher SCFA production from the grass diet and also no evidence that gelada cultures produced more SCFA from grass than from potato. There was also no evidence that greater SCFA concentrations due to diet were mediated by the presence of more bacteria, since dietary changes had no significant effect on total bacterial numbers. Also, although the SCFA concentration was weakly correlated with the total bacterial number (*t* = 2.4, *P* = 0.017), the relationship was not significant when the community and dietary effects outlined above were included in the model (*t* = 1.5, *P* = 0.13).

**FIG 4:**
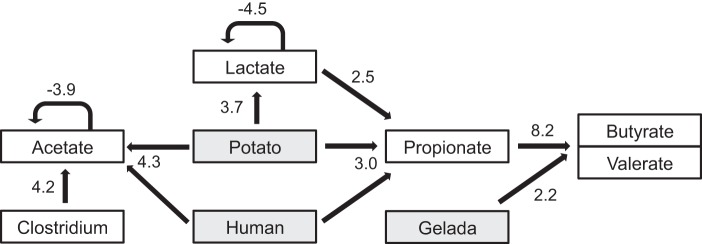
Summary of the results of linear models of production rates of SCFA depending on diet and community treatment (shaded boxes), SCFA concentration, and taxon counts (white boxes). Production rates were calculated as the changes in the concentration of each SCFA and lactate between time steps, and arrows show which variables at the initial time step correlate with those. Only terms retained as significant following simplification of linear models are shown, and numbers indicate the *t* value from linear models. Both acetate and propionate production rates were highest in human-derived cultures with the potato diet: the *t* value is for the interaction term between human and potato.

Inspecting the graphs for individual SCFA and lactate results shows an early peak of acetate and lactate production followed by propionate production followed by butyrate and valerate production (see Fig. S3 in the supplemental material). To investigate possible conversion of SCFA, we calculated the change in the concentration of each SCFA between time steps divided by the amount of time to give a rate of change in concentration. We then modeled how this rate depended on concentrations of SCFA and densities of each bacterial taxon at the preceding time step and on the culture community and diet and how it changed during the experiment (Fig. 4). Acetate production declined as the concentration of acetate increased (*t* = −3.9, *P* = 0.0002), consistent with a decline as the substrates being fermented to acetate were consumed. The production rate was higher in human cultures grown supplemented with grass (*t* = 2.2, *P* = 0.03) and especially potato (*t* = 4.3, *P* < 0.0001) and when the count of clostridium groups I and II was higher (*t* = 4.2, *P* < 0.0001). Lactate production rate also declined as lactate accumulated (*t* = −4.5, *P* < 0.0001). The rate was higher on the potato diet in both the human-derived and gelada-derived communities (*t* = 3.7, *P* = 0.0004). The propionate production rate was higher in the human-derived cultures on the potato diet (*t* = 3.0, *P* = 0.004) and depended on the concentration of lactate at the previous time point (*t* = 2.5, *P* = 0.013). We hypothesize therefore that lactate was being converted to propionate. The production rate of butyrate and valerate depended mainly on the concentration of propionate at the previous time step (*t* = 8.2, *P* < 0.0001) and was higher in the gelada-derived cultures than in human-derived cultures (*t* = 2.2, *P* = 0.034). Human cultures mostly had decreasing butyrate concentrations over time. Isovalerate concentrations were low and not predicted by any other variable.

To summarize, acetate and lactate were produced initially and more so on a potato diet, especially in the human cultures. The timing of changes in concentrations was consistent with lactate being converted to propionate, which in turn was partly converted to butyrate and valerate. Relatively more butyrate and valerate were produced in gelada cultures than in human cultures per mole propionate. The only bacterial effect on acid concentrations was that acetate production was higher with a higher density of clostridium groups I and II. These patterns could also be due to other hidden covariates, such as additional taxa that we did not count or additional metabolites that we did not measure.

### Effects of community, diet, taxonomic composition, and SCFA on PYY.

**FIG 5:**
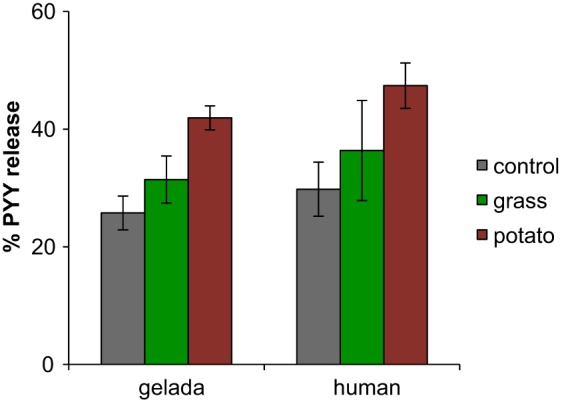
PYY release from colon cells exposed to effluent from *in vitro* microbial cultures with respect to the source community (gelada versus human) and the diet (control versus grass versus potato). To control for variation in the number of L cells per well, PYY release is expressed as follows: [(the amount of PYY measured in the supernatant)/(the amount of PYY measured in the supernatant plus lysed cells)] × 100. Results for samples from 0 and 10 h were pooled for each treatment, as time had no significant effect.

The production of PYY from colon cells *in vitro* was measured following exposure to effluent from cultures using a radioimmunoassay (RIA). PYY release depended primarily on diet (*F*_2,28_ = 17.7, *P* < 0.0001). One-third more PYY was released on the potato diet than on the grass diet (Fig. 5). Human cultures released 16% more PYY than geladas, but this result was not statistically significant. There was no evidence for an increase in PYY release from samples at later time points, suggesting that the dietary factors driving variations in PYY release were present from the outset. PYY release also increased with lower densities of lactobacilli (*t* = −4.1, *P* = 0.0032), increased with the concentration of butyrate (*t* = 2.9, *P* = 0.007), and decreased with the concentration of propionate (*t* = −2.2, *P* = 0.035). Note, however, that neither the butyrate results nor the propionate results were significant when included in a univariate regression and that they achieved significance only when they were included together in a multivariate regression: neither considered alone was a significant predictor of PYY release. The main effects of diet identified above therefore appear to act separately from intermediate effects on SCFA concentrations.

### Metabolic differences between human-derived and gelada-derived batch cultures and their response to diet.

**FIG 6:**
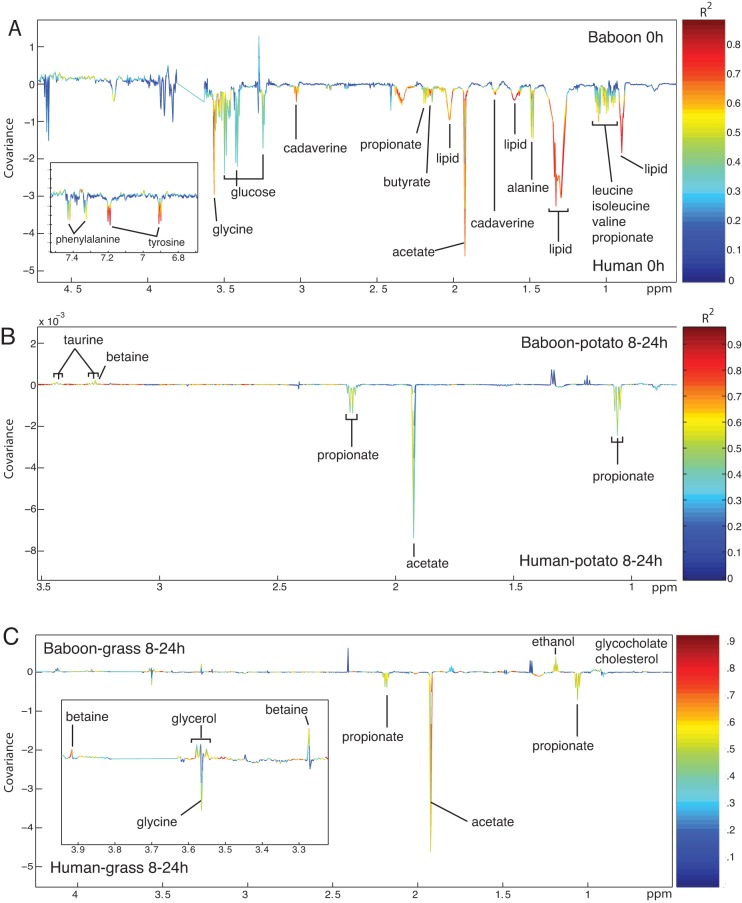
Plot of LS-DA coefficients comparing the metabolic profiles of human and gelada batch cultures at 0 h (*P* = 4.03 × 10^−4^) (A), 8 to 24 h following potato supplementation (B), and 8 to 24 h following grass supplementation (C).

The metabolic profiles of the batch cultures were characterized by ^1^H NMR spectroscopy. To identify basal metabolic variation between the species, partial least-squares-discriminant analysis (PLS-DA) was applied to the metabolic profiles of the human and gelada batch cultures at time 0 (*P* = 8.41 × 10^−4^; Fig. 6A; see also Fig. S4 in the supplemental material). Human batch cultures contained greater amounts of amino acids, namely, alanine, glycine, tyrosine, phenylalanine, leucine, isoleucine, and valine, than gelada cultures. Cadaverine, glucose, lipids, and the SCFA acetate, propionate, and butyrate were also present in greater amounts in the human cultures (see Fig. 6A in the supplemental material).

In the gelada-derived cultures, control and potato diets induced similar changes in metabolite concentrations (see Fig. S5 in the supplemental material): acetate, butyrate, propionate, ethanol, and trimethylamine levels increased over time while alanine, betaine, choline, taurine, and glycine levels decreased. In the grass-inoculated cultures, acetate, butyrate, ethanol, and trimethylamine levels increased while betaine and choline levels decreased over time. Succinate and glycine also increased in grass-inoculated vessels over time while trans-aconitate decreased. Across all treatments, metabolic profiles did not change between the 0- and 5-h sampling points. At 8 to 24 h, the potato-inoculated vessels contained larger amounts of ethanol, lactate, and formate than the grass-inoculated cultures. In contrast, grass-inoculated cultures contained higher levels of leucine, isoleucine, valine, alanine, betaine, trans-aconitate, and phenylalanine.

The metabolic profiles of human-derived cultures were less homogeneous among replicates than in gelada-derived cultures, leading to low significance for models relating metabolite composition to response variables. The control and potato-inoculated cultures displayed no significant changes in metabolite composition over time (*P* = 0.32 and *P* = 0.12, respectively). Heterogeneity across replicates and time points most likely explains this result. There were significant changes over time in the grass-inoculated cultures: acetate, butyrate, propionate, trimethylamine, phenylacetate, and isovalerate levels increased over time while choline, tyrosine, phenylalanine, alanine, and trans-aconitate levels decreased (see Fig. S6 in the supplemental material). A pairwise comparison of the 8-to-24-h metabolic profiles from the potato- and grass-inoculated human cultures revealed that those receiving potato had higher levels of propionate and acetate, whereas the grass-inoculated cultures contained greater levels of betaine, trans-aconitate, alanine, phenylalanine, and branched-chain amino acids: these profiles are similar to those of the gelada cultures (see Fig. S7).

Comparing human and gelada cultures, at 8 to 24 h, human vessels inoculated with the potato diet contained more acetate and propionate and gelada vessels contained higher taurine and betaine levels (Fig. 6B). At that time point, human cultures inoculated with the grass diet contained larger amounts of glycine, acetate, and propionate, whereas gelada cultures contained greater amounts of formate, ethanol, betaine, glycerol, cholesterol, and glycocholate (Fig. 6C). Compared to baseline levels, these results indicate that grass stimulated the gelada microbiota to produce ethanol and glycerol while it stimulated the human microbiota to produce acetate and propionate.

### Metabolic correlates of PYY release.

**FIG 7:**
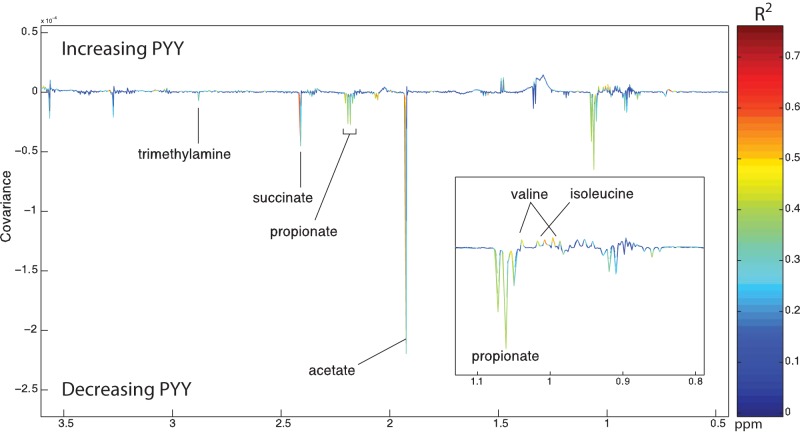
PLS model identifying metabolic correlates of PYY release in effluent from each culture. Communities, diets, and times were pooled for the analysis.

To identify metabolites correlated with PYY secretion, PLS models were built using PYY release as the response variable. PYY release correlated negatively with trimethylamine and propionate levels and positively with valine and isoleucine levels (Fig. 7). We repeated the regression model analyses predicting PYY release outlined above but included additional variables identified by NMR as potential predictors. This led to a simplified model with isoleucine (*t* = 2.8, *P* = 0.011), betaine (*t* = −3.3, *P* = 0.0024), lactobacilli (*t* = −3.8, *P* = 0.0007), and diet (*F*_2,18_ = 10.3, *P* = 0.001).

## DISCUSSION

We found that dietary differences played the leading role in determining the metabolism and effects on gut hormone production across gut microbial cultures. Human-derived cultures initially contained more total bacteria and more of all the study bacterial taxa than gelada-derived cultures. Indeed, the gelada fecal samples were green with little odor and resembled partly digested grass. A study of fermentation of hay by gelada feces compared to zebra and hamadryas baboon feces also found fermentation by the gelada bacterial community to be less effective than expected (19). Perhaps as a result of this, human microbiota metabolized the diets faster than the gelada microbiota and with higher peak production of SCFA. However, total bacteria in gelada samples increased to be equivalent after 24 h. Both microbiota were clearly capable of metabolizing both diets, which indicates flexibility rather than strict specialization to accustomed diets (being higher starch in a human vegetarian diet and consisting mainly of grass for the preceding summer months in the geladas). In the gelada baboon cultures, potato supplementation led to greater consumption of bacterial substrates (phenylalanine, alanine, betaine, and trans-aconitate) and higher production of bacterial metabolites (ethanol, lactate, acetate, and formate) at 8 to 24 h than grass supplementation. This implies that potato-stimulated bacterial metabolism is quicker than that associated with grass in both communities.

Of course, geladas and humans have other adaptations to their differing diets not investigated here. For example, geladas have a specialized masticatory apparatus that allows them to reduce foods to small particle sizes, which increases energy extraction (22). They also have relatively large colons and ceca, although these are not much different from those of other baboons that do not specialize on grass (19). Observation of feeding times suggests that geladas do not have long transit times to compensate for the relatively low quality of their diets but instead have a high-volume, rapid-transit strategy similar to that of horses (23).

The main taxonomic shift that we observed during the experiment was that clostridium groups I and II, lactobacilli, and, to a lesser extent, bifidobacteria all declined in density in the human-derived cultures and fastest on the potato diet. There is evidence for an initial pulse in growth of these taxa before a dramatic decline: this likely reflects their dependence on starch-based polymers and monomers that were subsequently used up and their ability to use up substrate rapidly. This interpretation is supported by the observation of an initial peak in levels of acetate and lactate, which these taxa produce as end products. Lactate appeared to be subsequently converted to propionate and butyrate, a conversion which can be carried out by other members of the gut microbial community such as *Eubacterium rectale* (24), which was present at high densities in the human-derived cultures. *Bacteroides* species tend to be generalists and can also convert lactate to propionate and utilize additional polymers aside from starch, such as pectin and xylans (25).

We chose FISH to focus on taxa of particular interest and to obtain numerical counts. It is possible that we missed interesting changes in other taxa, especially in the gelada communities, since the FISH probes were designed for use with human microbiota. Future use of 16S rRNA gene sequencing would be interesting to check for additional taxa that might have varied in their responses. However, for our aims of identifying correlates of SCFA and PYY production, there would have been limits to how many taxa we could have incorporated into our statistical models, set by the amount of replication we could implement for the experiments (as a rule of thumb, three times more data points are needed than explanatory variables). Note also that, over the longer term, the effects of switching to a new diet could be ameliorated by colonization of new bacteria from the environment. Our experiments looked at instantaneous effects of feeding gut communities different foods.

We found no evidence that SCFA production was enhanced by the grass diet with high levels of plant cell wall fiber: the highest levels were obtained on the potato diet in both the human-derived and gelada-derived cultures. Note that we added equal amounts of digested material for the two diets, and so these results do not reflect larger amounts of indigestible material but that the type of material in the potato diet—presumably mainly resistant starch—could be metabolized more readily into SCFA than the material in the grass diet (presumably mainly cellulose and lignin). High acetate production rates were found with higher *Clostridium* groups I and II counts, consistent with their role in degrading starch- and cell wall-derived polymers (25).

The human cultures displayed a greater effect of diet than the gelada cultures, which were unable to produce as much SCFA as human cultures or to produce SCFA as rapidly and showed lesser differences in the production rates of the two diets. Greater SCFA production occurred with the microbiota derived from humans than with those obtained from geladas even on the grass diet. The one “positive” effect of the gelada microbiota was a higher inferred rate of conversion from propionate to butyrate (although butyrate concentrations were still higher in the human cultures than in the gelada cultures). Overall, this indicates that the gelada microbiota is not specialized to release SCFA from their grass-based diet. After 72 h, there was no significant effect of the source community on SCFA concentrations, despite the radically different natures of the two diets. Although grass did not stimulate increased SCFA production according to a naive ancestral-diet hypothesis, our results demonstrate remarkable flexibility of modern human microbiota: from our findings, it appears that modern-day vegetarians could still extract some energy from grass. Also, with regard to the ancestral-diet hypothesis, although it is likely that ancestral diets were high fiber, some might have been high starch as well (cf. reference 26). One possible limitation for our approach is that SCFA production in batch cultures might differ from production *in vivo* if feedback mechanisms affect production rates: gut epithelial cells remove SCFA from real guts, but SCFA were free to accumulate in our cultures. We did control pH in the cultures to remove possible feedback effects from acidification caused by metabolism.

PYY results broadly mirrored those for SCFA: the release depended mainly on diet and was higher on the potato diet than the grass diet. However, there was no simple predictive relationship between SCFA concentrations and the levels of PYY release across samples. Acetate concentration was correlated with PYY release as a single predictor, but this relationship was lost when diet itself was included. This indicates that some other aspect of diet might be a stronger determinant. There was evidence that high concentrations of butyrate relative to propionate (which we observed to be potentially linked by conversion above) stimulated PYY release, which would fit with stronger effects of butyrate on colon cells (27). However, again, this was far less predictive than diet itself. Regarding the effects of the focal taxa on PYY release, we found a strong relationship between lower densities of lactobacilli and higher PYY release that was unexpected. One possible explanation is that lactobacilli use up substrate but only to produce lactate, which might divert resources away from production of molecules triggering the FFAR2/3 receptors by other taxa. However, as argued below, a broader survey of metabolites is needed to identify the metabolites with the strongest effects on the signaling pathway.

Prospecting for additional effects of our treatments and triggers of PYY release through NMR spectroscopy revealed some additional potential correlates. The human and gelada cultures again mainly differed in the speed of response: human cultures quickly reached a stable metabolite composition, whereas geladas exhibited slower linear changes over the 72 h. Most interestingly, three metabolites were identified that displayed stronger correlations with variations in PYY release across cultures than were seen with SCFA. These were isoleucine and valine, closely correlated with each other, which are amino acids commonly used as appetite suppressant supplements, and betaine, which again might reflect metabolism of protein rather than polysaccharides or fiber and correlated negatively with PYY release. These signals would require further tests with single-metabolite exposure to verify their causal effect on PYY release, but they point toward fermentation products of protein in diets as playing a greater role in appetite suppression or stimulation than those from resistant starch and other dietary fibers. However, even including these additional metabolites, the main correlate of PYY release was diet *per se* rather than chemical composition in the cultures as measured here. Functionally, it might be hypothesized that low PYY release on a nutrient-poor grass diet might allow continued feeding without triggering appetite suppression hormones.

In conclusion, we have confirmed that diet plays a major role in the interaction between bacterial metabolism and gut hormone signaling. Within the space of 72 h, there was remarkable convergence between metabolism and effects on hormone release of microbiota sourced from two primates with very different diets. However, the complexity of these systems makes it difficult to take simple predictions from single taxon and metabolite studies and apply them to outcomes in whole feces-derived communities. Our results were more complex than those predicted from the wealth of studies on effects of SCFA. For example, there was an effect of butyrate on PYY release but only as an increasing butyrate/propionate ratio and subsidiary to a bigger unspecific effect of diet. More manipulative experiments on whole microbiota are needed, with further resolution of taxonomic and metabolic effects, to resolve these issues.

## MATERIALS AND METHODS

### Preparation of grass- and potato-supplemented media.

*Festuca rubra* grass was collected from the Harris gardens at the University of Reading. The fresh grass was cut into 1-cm-long pieces prior to predigestion. Potatoes were finely grated in preparation for simulated *in vitro* human digestion. Predigestion of the samples was performed according to the method described in reference 28. Four 60-g portions of each grass and potato sample were ground, and then 101 ml of distilled water was added. The mixture was then processed with a stomacher device for 5 min. The oral phase was simulated by mixing with 20 mg α-amylase (A 4551; Sigma)–6.25 ml CaCl_2_ (0.001 M, pH 7.0). This was then incubated at 37°C for 30 min on a shaker. HCl (6 M) was used to lower the pH to 2.0. For the gastric phase, 2.7 g of pepsin (P 7000; Sigma) was dissolved in 25 ml HCl (0.1 M), added to the samples, and incubated at 37°C for 2 h on a shaker. For the small-intestine phase, 560 mg of pancreatin (P 8096; Sigma) and 3.5 g of bile (B 8631; Sigma) were dissolved in 125 ml of NaHCO_3_ (0.5 M). The pH was adjusted to 7.0 using 6 M HCl or 6 M NaOH. The solution was then incubated at 37°C for 3 h. The sample solution was then transferred to 1-kDa-cutoff cellulose dialysis tubing (Spectra/Por 6; Spectrum Europe, Netherlands) and dialyzed against 0.01 M NaCl at 5°C. This was done in order to remove low-molecular-mass digestion products. After 15 h, the dialysis fluid was changed and dialysis continued for a further 2 h. Following this, samples were freeze-dried for 5 days, ready for use in *in vitro* fermentation.

### Fecal batch cultures.

Sterile stirred-batch culture fermentation vessels (100 ml working volume) were filled with 45 ml of sterile basal nutrient medium (starch [5 g], peptone water [5 g], tryptone [5 g], yeast extract [4.5 g liter^−1^], NaCL [4.5 g liter^−1^], KCl [4.5 g liter^−1^], mucin [4 g liter^−1^], casein [3 g liter^−1^], pectin [2 g liter^−1^], xylan [2 g liter^−1^], arabinogalactan [2 g liter^−1^], NaHCO_3_ [1.5 g liter^−1^], MgSO_4_ [1.25 g liter^−1^], guar gum [1 g liter^−1^], inulin [1 g liter^−1^], cysteine hydrochloride [0.8 g liter^−1^], KH_2_PO_4_ [0.5 g liter^−1^], K_2_HPO_4_ [0.5 g liter^−1^], bile salts [0.4 g liter^−1^], CaCl_2 ⋅_ 6H_2_O [0.15 g liter^−1^], FeSO_4 ⋅_ 7H_2_O [0.005 g liter^−1^], hemin [0.05 g liter^−1^), Tween 80 [1 ml liter^−1^], vitamin K [10 µl liter^−1^], resazurin [0.0001 g liter^−1^], pH 4). All chemicals were purchased from Oxoid and Sigma. Anaerobic conditions were maintained by sparging the vessels with O_2_-free N_2_ (15 ml min^−1^) overnight. Temperature was held at 37°C using a circulating water bath, and pH was controlled between 6.7 and 6.9 using an automated pH controller (Fermac 260; Electrolab, Tewkesbury, United Kingdom) which added acid and alkali as required (0.5 M HCl and 0.5 M NaOH). For the human gut communities, a fecal slurry from three healthy vegetarian donors, who had taken no prebiotics, probiotics, or antibiotics within 3 months before the study, was prepared with phosphate-buffered saline (PBS) (to yield a 10% [wt/vol] fecal slurry). We chose vegetarians because we were comparing two plant-based foods and wanted to compare gelada communities to those from humans with a plant-based diet. Freshly voided feces of 3 gelada baboon individuals were collected from Howletts Wild Animal Park in Kent in September 2011 and transferred in anaerobic flasks within 2 h to set up fecal slurrys in the same way. The geladas live in a large grass paddock and mainly feed on grass over the summer. Each vessel was inoculated with 5 ml of freshly prepared fecal slurry prepared from an individual donor. Predigested potato, grass, or no substrate (control) was added to the batch cultures, which were processed in triplicate. Batch cultures were run for 72 h and samples obtained from each vessel at 0, 5, 8, 24, 48, and 72 h for enumeration of bacterial populations using FISH as described in reference 29. We chose 72 h to represent a long transit time (the average for humans is 48 h, but 72 h is not uncommon). Although in reality individuals eat multiple times during this period, material passes along the gut and so addition of further food is not relevant to the progress of metabolism within a given sample of material. Also, we monitored changes over shorter time scales, where most differences were found; hence, our results were not dependent on the 72-h duration. Probes were commercially synthesized and 5′-labeled with fluorescent Cy3 dye (Sigma-Aldrich, St. Louis, MO; see Table S1 in the supplemental material). Another sample set was centrifuged at 13,000 × *g*, and the supernatant was stored at −20°C for SCFA analysis. Samples for metabolomic analysis were frozen immediately for use in a primary colonic cell digest to assess levels of PYY and GLP-1. Furthermore, the fermentation supernatant was also tested for radioimmunoassay (RIA; see below).

### Gas chromatography.

The short-chain fatty acids acetate, propionate, butyrate, isobutyrate, valerate, isovalerate, and caproate were analyzed as their salyl derivatives by gas chromatography using the exact protocol described in reference 30. The sample was run on a 5890 series II GC system (HP, Crawley, West Sussex, United Kingdom) fitted with a SGE-HT5 column (0.32 mm by 25 m; 0.1 µm pore size) (J and W Scientific) and a flame ionization detector. Peaks were integrated using Atlas Lab managing software (Thermo Lab Systems, Mainz, Germany). SCFA concentrations were calculated in mmole per liter by comparing their peak areas with standards.

### ^1^H NMR spectroscopy.

Batch culture samples (400 µl) were combined with 200 µl of phosphate buffer (pH 7.4; 100% D_2_O) containing 1 mM of the internal standard, TSP (3-trimethylsilyl-1-[2,2,3,3-^2^H4] propionate). Samples were mixed by vortex and centrifuged (10,000 × *g* for 10 min) before transfer to a 5-mm-diameter NMR tube. Spectroscopic analysis was carried out on a 700-MHz Bruker NMR spectrometer equipped with a CryoProbe. Standard one-dimensional ^1^H NMR spectra of the batch culture samples were acquired with water peak suppression using a standard pulse sequence. For each sample, 8 dummy scans were followed by 128 scans and collected into 64,000 data points. A spectral width of 20 ppm was used. Chemical shifts in the spectra were referenced to the TSP singlet at δ 0.0. Spectra were manually phased and corrected for baseline distortions. The ^1^H NMR spectra (δ 0.2 to 10.0) were digitized into consecutive integrated spectral regions (~20,000) of equal widths (0.00055 ppm). The regions containing the residual signal from water (δ 4.50 to 5.00) and the large resonance arising from polyethylene glycol (δ 3.70) were removed to minimize the effects of baseline distortion. Each spectrum was then normalized using a probabilistic quotient approach (31). Multivariate modeling, including principal component analysis (PCA) and partial least-squares analysis (PLS) (32), was performed on the samples in MatLab (The Mathworks, Inc., Natwick, MA) using scripts provided by Korrigan Sciences Ltd., United Kingdom. The ^1^H NMR spectroscopic data were used as the descriptor matrix, and community type, sampling time (0, 5, 8, 24, 48, and 72 h), diet, and PYY release were successively used as the response variables. The contribution of each variable (metabolite) to the response was visualized by back-scaling transformation, generating a plot of correlation coefficients. Plots of coefficients are colored according to significance of correlation to time.

### Primary mouse colonic cell cultures.

Colonic cell cultures were prepared as described in reference 33. In brief, adult male C57BL6 mice (8 weeks of age or older) were culled and the colon, distal to the cecum, was removed, cleaned, and placed into ice-cold L-15 (Leibowitz) medium. The work was approved under schedule 1 of the British Home Office Animals (Scientific Procedures) Act of 1986. The intestinal tissue was digested with 0.4 mg/ml collagenase XI (Sigma, United Kingdom) in high-glucose Dulbecco’s modified Eagle’s medium (DMEM) (Sigma, United Kingdom) at 37°C. The resulting cell suspensions were centrifuged, and the pellets were resuspended in DMEM (supplemented with 10% fetal calf serum and 1% antibiotics, 100 U/ml penicillin, and 0.1 mg/ml streptomycin). The combined cell suspensions were filtered through a nylon mesh (pore size, 250 µm) and plated onto 24-well, 1% Matrigel-coated plates. The plates were incubated overnight at 37°C in an atmosphere of 95% O_2_ and 5% CO_2_.

### Secretion experiments.

PYY secretion experiments were carried out within 24 h after plating. The cells were washed three times with secretion buffer (4.5 mM KCl, 138 mM NaCl, 4.2 mM NaHCO_3_, 1.2 mM NaH_2_PO_4_, 2.6 mM CaCl_2_, 1.2 mM MgCl_2_, and 10 mM HEPES, which was adjusted to pH 7.4 with NaOH) supplemented with 0.1% fatty-acid-free bovine serum albumin (BSA). The cells were then incubated in secretion buffer containing the diluted effluents for 2 h at 37°C in an atmosphere of 95% O_2_ and 5% CO_2_. Following incubation, the cell supernatants were collected, centrifuged at 4°C, and stored at −20°C. The cells remaining in the plates were treated with cell lysis buffer and were frozen at −80°C overnight. The plates were then scraped and washed using secretion buffer, and lysates were stored at −20°C pending analysis.

### PYY radioimmunoassay.

PYY-like immunoreactivity was measured using a specific and sensitive radioimmunoassay (34). The antiserum (Y21) was produced in rabbits against synthetic porcine PYY coupled to BSA by glutaraldehyde. The Y21 antibody cross-reacts fully with the biologically active forms of PYY: full-length PYY_1-36_ and the truncated fragment PYY_3-36_. It does not cross-react with other gastrointestinal peptides.^125^ I–PYY was prepared using the iodogen method (35) and purified by high-performance liquid chromatography (HPLC). The assay was performed in a total volume of 350 µl of 0.06 M phosphate buffer (pH 7.3) containing 0.3% BSA. The standard curve was constructed by adding 1, 2, 3, 5, 10, 15, 20, 30, 50, and 100 µl of synthetic PYY at a concentration of 0.5 pmol/ml. The assay was incubated over three nights at 4°C before separation of the free label from the antibody-bound label by immunoprecipitation using sheep anti-rabbit antibody. Free radioactivity and bound radioactivity were measured using γ scintillation counters.

## SUPPLEMENTAL MATERIAL

Figure S1 Bacterial densities in the human- and gelada-derived cultures at the start of the experiment. Standard error bars are shown. Levels of lactobacilli were not significantly different between communities (*t* = 1, *P* = 0.3); levels of all other taxa were significantly higher in human communities (all *t* > 3, all *P* < 0.005). Download Figure S1, EPS file, 0.7 MB

Figure S2 The first two principal components representing variation in the taxonomic composition. Each point represents a single culture at a single time point. PC1 and PC2 explain 43.0% and 37.0% of the variations in composition, respectively. PC1 represents increasing *Bacteroides*, *E. rectale*, and bifidobacteria densities and is significantly associated with the source community. PC2 represents decreasing densities of Clostridium groups I and II and lactobacilli and decreases significantly over time, especially in human-derived cultures grown on potato. Analyses were conducted using the prcomp function in the R statistical programming language. Download Figure S2, EPS file, 0.4 MB

Figure S3 Concentrations of SCFA and lactate through time. Colors indicate diet: black = control; green = grass; brown = potato. Solid lines = gelada cultures. Dashed lines = human cultures. Standard errors are shown. Download Figure S3, EPS file, 0.3 MB

Figure S4 An example of 700-MHz ^1^H NMR spectra measured from human (control, 0 h) (A) and baboon (control, 0 h) (B) batch culture samples. PEG, polyethylene glycol. Download Figure S4, EPS file, 2.4 MB

Figure S5 PLS models identifying time-dependent shifts in the metabolic profiles of gelada cultures over 72 h exposed to control (A), potato (B), and grass (C) diets. Download Figure S5, TIF file, 0.6 MB

Figure S6 PLS models identifying time-dependent shifts in the metabolic profiles of human cultures exposed to grass diets over 72 h (*P* = 0.012). Download Figure S6, TIF file, 0.4 MB

Figure S7 Plots of LS-DA coefficients comparing the metabolic responses of cultures to potato and grass diets at 8 to 24 h. Data represent baboon (*P* = 0.007) (A) and human (B) cultures (*P* = 0.014). BCAA, branched-chain amino acids. Download Figure S7, TIF file, 0.6 MB

Table S1 Probes used for FISH analysis of bacterial populations in samples from batch culture systems. Table S1, PDF file, 0.1 MB.
