# Supplementary figures and images for: Impacts of Plant-Based Foods in Ancestral Hominin Diets on the Metabolism and Function of Gut Microbiota In Vitro
Source: mBio. 2014 May 20;5(3):e00853-14. doi: 10.1128/mBio.00853-14 (PMC4030449; doi:10.1128/mBio.00853-14)

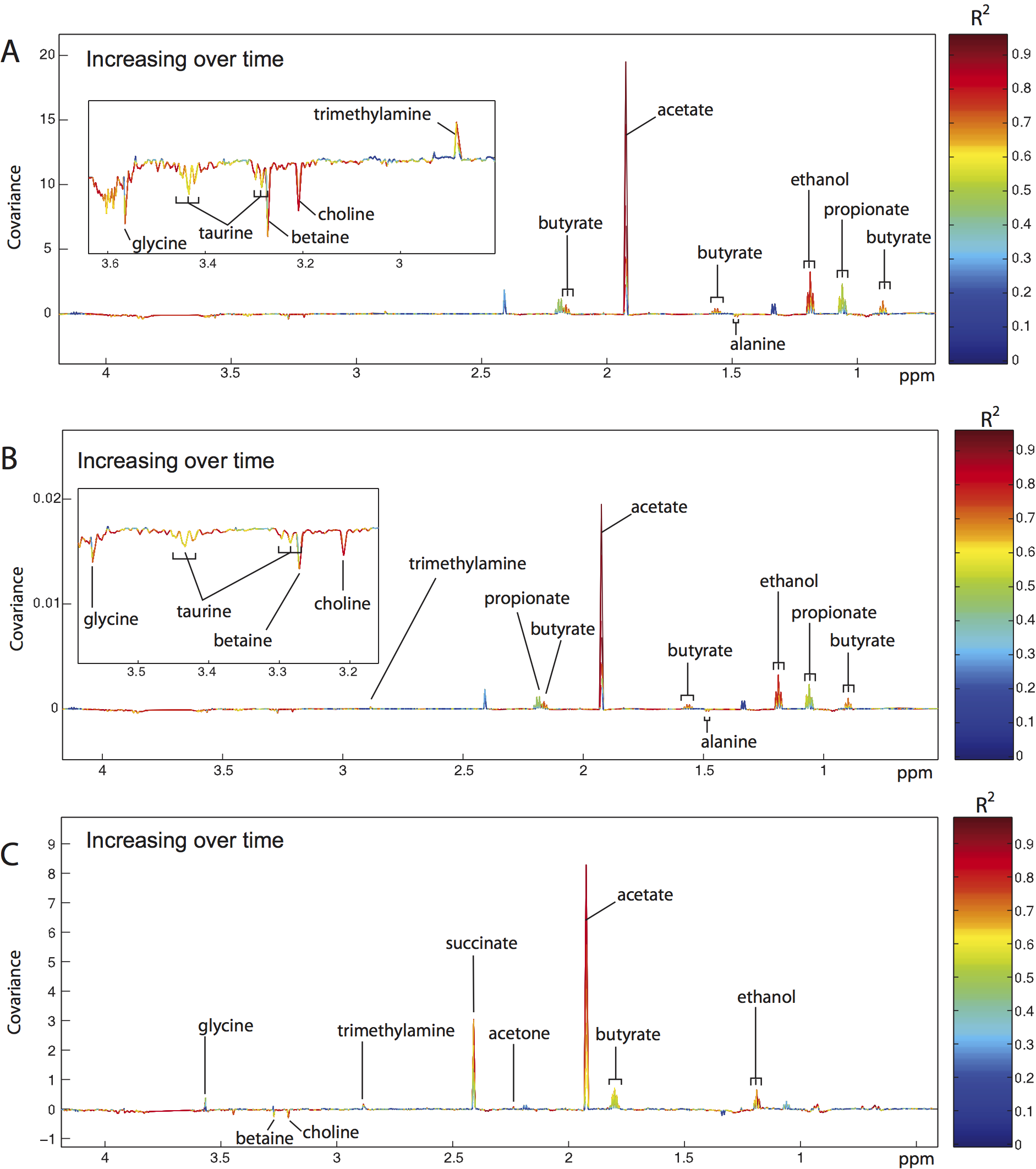

Supplement: Figure S5 — PLS models identifying time-dependent shifts in the metabolic profiles of gelada cultures over 72 h exposed to control (A), potato (B), and grass (C) diets. Download [file mbo003141837sf05.tif]

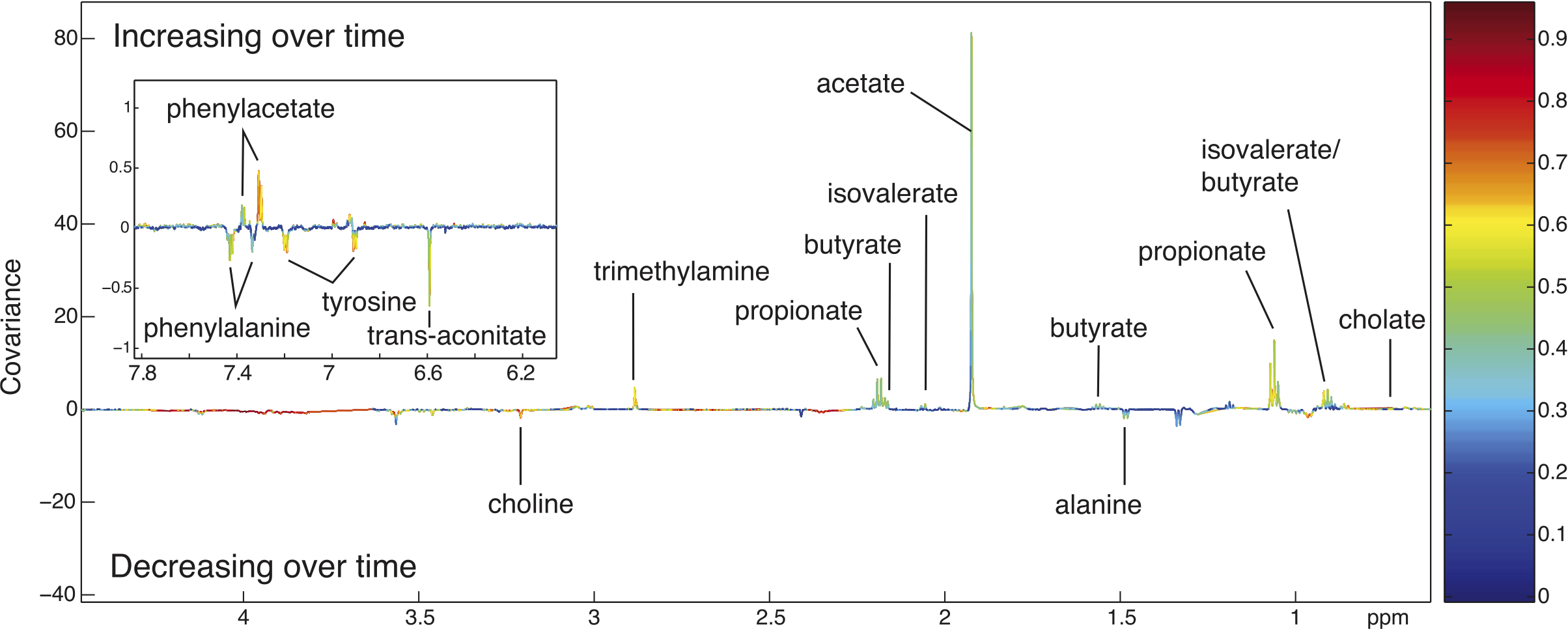

Supplement: Figure S6 — PLS models identifying time-dependent shifts in the metabolic profiles of human cultures exposed to grass diets over 72 h (P = 0.012). Download [file mbo003141837sf06.tif]

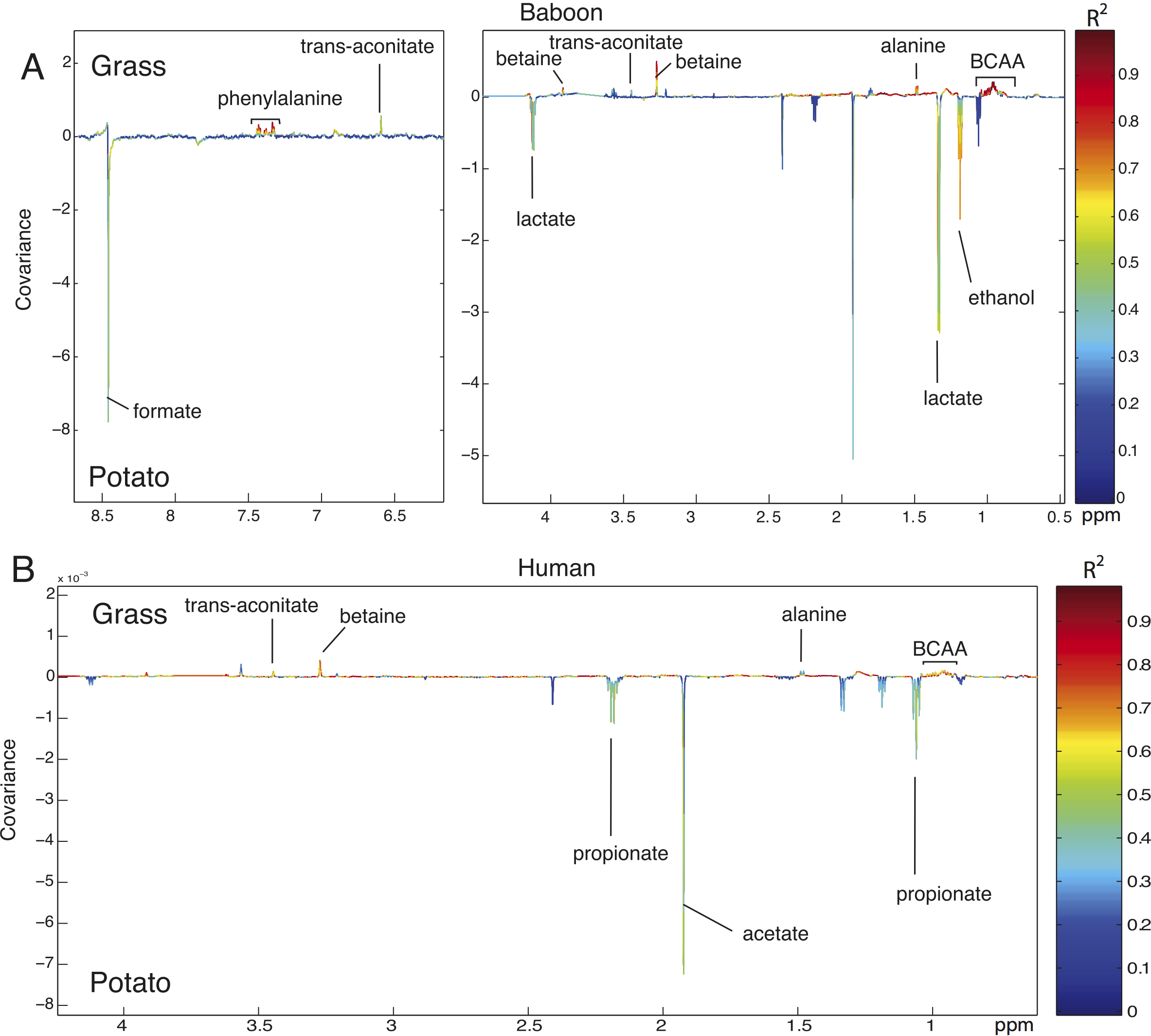

Supplement: Figure S7 — Plots of LS-DA coefficients comparing the metabolic responses of cultures to potato and grass diets at 8 to 24 h. Data represent baboon (P = 0.007) (A) and human (B) cultures (P = 0.014). BCAA, branched-chain amino acids. Download [file mbo003141837sf07.tif]
