# Supplementary material for: Impacts of Plant-Based Foods in Ancestral Hominin Diets on the Metabolism and Function of Gut Microbiota In Vitro
Source: mBio. 2014 May 20;5(3):e00853-14. doi: 10.1128/mBio.00853-14 (PMC4030449; doi:10.1128/mBio.00853-14)
Supplement: Table S1 — Probes used for FISH analysis of bacterial populations in samples from batch culture systems. [file mbo003141837st1.pdf]

Table S1. Probes used for FISH analysis of bacterial populations in samples from batch culture systems

Short name	Accession no.*	Full name†	Target species	Temperature		Sequence (5´ to 3´)	Reference
				Hybridisation	Washing		
Bif164	pB-00037	S-G-Bif-0164-a-A-18	Most *Bifidobacterium* spp. and *Parascardovia denticolens*	50	50	CATCCGGCATTACCACCC	Langendijk et al.,1995
Lab158	ND	S-G-Lab-0158-a-A-20	Most *Lactobacillus, Leuconostoc* and *Weissella* spp.; *Lactococcus lactis*; all *Vagococcus, Enterococcus, Melisococcus, Tetragenococcus, Catellicoccus, Pediococcus* and *Paralactobacillus* spp.	50	50	GGTATTAGCAYCTGTTTCCA	Harmsen et al., 1999
Bac303	pB-00031	S-*-Bacto-	Most *Bacteroides sensu stricto* and	46	48	CCAATGTGGGGGACCTT	Manz et al., 1996

		0303-a-A-17	*Prevotella* spp.; all *Parabacteroides*; *Barnesiella viscericola* and *Odoribacter splanchnicus*				
Chis150	pB-00962	S-*-Chis-0150-a-A-23	Most members of *Clostridium* cluster I; all members of *Clostridium* cluster II; *Clostridium tyrobutyricum*; *Adhaeribacter aquaticus* and *Flexibacter canadensis* (family *Flexibacteriaceae*); [*Eubacterium*] *combesii* (family *Propionibacteriaceae*)	50	50	TTATGCGGTATTAATCTYCCTTT	Franks et al., 1998
Erec482	pB-00963	S-*-Erec-0482-	Most members of *Clostridium* cluster XIVa; *Syntrophococcus*	50	50	GCTTCTTAGTCARGTACCG	Franks et al., 1998

	a-A-19	*sucromutans*, [*Bacteroides*] *galacturonicus* and [*Bacteroides*] *xylanolyticus*, *Lachnospira pectinschiza* and *Clostridium saccharolyticum*				
Eub I		Most bacteria	46	48	GCTGCCTCCCGTAGGAGT	Harmsen et al., 1999
Eub II					GCTGCCACCCGTAGGTGT	
Eub III					GCAGCCACCCGTAGGTGT	

*ND, No information relating to these probes has been deposited in probeBase (http://www.microbial-ecology.net/probebase).

†Probe designation according to Alm et al. (1996). This information was retrieved from probeBase.

‡These probes were used together in equimolar concentrations (both at 50 ng μl^{-1}). Formamide (20 %) was included in the hybridization buffer.
